# FINANCIAL EXPLOITATION AMONG HOLOCAUST SURVIVORS: EVIDENCE FOR VULNERABILITY AND RESILIENCE

**DOI:** 10.1093/geroni/igae098.0576

**Published:** 2024-12-31

**Authors:** Gali Weissberger, Moshe Bensimon, Amit Shrira

**Affiliations:** Bar-Ilan University, Ramat Gan, HaMerkaz, Israel; Bar-Ilan University, Ramat Gan, HaMerkaz, Israel; Bar-Ilan University, Ramat Gan, HaMerkaz, Israel

## Abstract

Late-life adverse events may be particularly impactful in Holocaust survivors (HS) as they may be reminiscent of certain conditions that existed during the Holocaust. Financial exploitation (FE) is one such adverse event that may lead to losses reminiscent of Holocaust conditions. Research has demonstrated that the experience of FE has devastating consequences on the mental health of older adults. However, few research studies have examined the consequences of FE in older adults with a traumatic past. In a quantitative study, we showed that anxiety and depressive symptoms associated with an FE experience were significantly greater in HS with posttraumatic stress disorder (PTSD) symptoms relative to comparisons (HS without PTSD symptoms who experienced FE and HS without an FE experience). However, research of HS has also shown that they exhibit certain resiliencies vis-à-vis stress. Accordingly, we used a qualitative approach to further examine vulnerability and resilience among HS who experience FE. The following questions guided our inquiry: What are the feelings and perceptions of HS following an experience of FE? How did they cope with FE? What are their perceptions of protective and risk factors for FE? Fifteen HS (10 females, M age = 84.66) living in Israel underwent semi-structured interviews. A four-stage content analysis revealed several core themes of meaning: 1) negative emotional reactions towards FE; 2) coping mechanisms; 3) positive life motto; 4) protective and risk factors for FE. Taken together, the qualitative analyses produced novel insights with regards to vulnerabilities and resiliencies amongst HS who have experienced FE.

